# Deep Complex Gated Recurrent Networks-Based IoT Network Intrusion Detection Systems

**DOI:** 10.3390/s24185933

**Published:** 2024-09-13

**Authors:** Engy El-Shafeiy, Walaa M. Elsayed, Haitham Elwahsh, Maazen Alsabaan, Mohamed I. Ibrahem, Gamal Farouk Elhady

**Affiliations:** 1 Department of Computer Science, Faculty of Computers & Artificial Intelligence, University of Sadat City, Sadat City 32897, Egypt; 2 Department of Information Technology, Faculty of Computers & Information Systems, Damanhour University, Damanhour 22511, Egypt; walaazaid@cis.dmu.edu.eg; 3 Computer Science Department, Faculty of Computers and Information, Kafrelsheikh University, Kafrelsheikh 33516, Egypt; 4 Department of Computer Engineering, College of Computer and Information Sciences, King Saud University, Riyadh 11543, Saudi Arabia; malsabaan@ksu.edu.sa; 5 School of Computer and Cyber Sciences, Augusta University, Augusta, GA 30912, USA; mibrahem@augusta.edu; 6 Computer Science Department, Faculty of Computers and Information, Menoufia University, Shebin Elkom 32511, Egypt; gamal.farouk@ci.menofia.edu.eg

**Keywords:** deep neural learning, convolutional neural networks (CNN), internet of things (IoT), complex gated recurrent networks (CGRNs), anomaly detection, intrusion detection system (IDS)

## Abstract

The explosive growth of the Internet of Things (IoT) has highlighted the urgent need for strong network security measures. The distinctive difficulties presented by Internet of Things (IoT) environments, such as the wide variety of devices, the intricacy of network traffic, and the requirement for real-time detection capabilities, are difficult for conventional intrusion detection systems (IDS) to adjust to. To address these issues, we propose DCGR_IoT, an innovative intrusion detection system (IDS) based on deep neural learning that is intended to protect bidirectional communication networks in the IoT environment. DCGR_IoT employs advanced techniques to enhance anomaly detection capabilities. Convolutional neural networks (CNN) are used for spatial feature extraction and superfluous data are filtered to improve computing efficiency. Furthermore, complex gated recurrent networks (CGRNs) are used for the temporal feature extraction module, which is utilized by DCGR_IoT. Furthermore, DCGR_IoT harnesses complex gated recurrent networks (CGRNs) to construct multidimensional feature subsets, enabling a more detailed spatial representation of network traffic and facilitating the extraction of critical features that are essential for intrusion detection. The effectiveness of the DCGR_IoT was proven through extensive evaluations of the UNSW-NB15, KDDCup99, and IoT-23 datasets, which resulted in a high detection accuracy of 99.2%. These results demonstrate the DCG potential of DCGR-IoT as an effective solution for defending IoT networks against sophisticated cyber-attacks.

## 1. Introduction

Globally, the Internet of Things (IoT) is expanding quickly due to rising consumer expectations, a growing range of products, and advanced technology. Our everyday lives now involve billions of gadgets that are connected thanks to this proliferation; by 2023, Gartner predicts that there will be about 25 billion connected devices [1]. IoT technology offers advantages such as smarter solutions and improved daily activities, but these are outweighed by serious privacy and security issues. Managing a variety of linked devices, complexities, conflicting trends, and diversities is necessary while developing Internet of Things solutions. Conventional security measures are insufficient for the extended sessions that are common in Internet of Things contexts since they were created for powerful computers and brief sessions. These elements make Internet of Things (IoT) devices appealing targets for cybercriminals, putting our lives in danger. Developing efficient security solutions based on “lightweight” and “adaptive” concepts is a pragmatic way to handle IoT complexity. Large-scale distributed networks have shown success using “Adaptive Lightweight” techniques in managing anomalies. However, because there are so many IoT devices on a network, it is not feasible to develop unique security solutions for every item.

Securing data shared between devices in an IoT network, on the other hand, could be a realistic technique. To generate adaptive solutions for the IoT system, artificial intelligence can be utilized to analyze a wide range of data quantities and types. Current security techniques are only appropriate for powerful machines and short-term sessions, and the same protection strategy cannot be employed for long-running sessions. As a result, IoT devices have become appealing targets for hackers, placing our lives in danger from unknown risks. Designing effective security solutions based on the principles of “lightweight” and “adaptation” may be a viable method for handling the complexity of the IoT. “Adaptive Lightweight” approaches have proven helpful in dealing with abnormalities in big, distributed networks. The sheer quantity of IoT devices on a network makes developing a security solution for each one difficult. However, protecting the data transmitted between IoT devices could be a viable approach.

Artificial neural networks with layers of artificial neurons are subjected to deep learning. As data move from the first layer, known as the input layer, via intermediate (hidden) layers to the last layer, known as the output layer, it is processed in such a network. An artificial neural network that has multiple intermediate layers is referred to as deep.

The activation function is the one in which neurons are represented. The neuron’s output is the outcome of applying the activation function to the scalar product of the input vector and the neuron’s weight vector, shifted by a specified distance [2]. The activation function’s value is determined by the weighted sum of the neuron’s inputs and the threshold value. Useful instances of nonlinear functions are the sigmoid, SoftMax function, hyperbolic tangent, and rectified linear unit (ReLU), which are a few nonlinear functions that have been utilized as activation functions.

A loss function, often known as error, is used to describe the discrepancy between the target variable’s actual value and the value the neural network predicts during training.

Neural network topologies vary according to the arrangements of neurons, layers, and connections between them. Thus, the neural network design that is employed can be utilized to categorize deep learning techniques.

Furthermore, deep learning techniques can be categorized into four primary types, like classic machine learning techniques: supervised, semi-supervised, unsupervised, and reinforced deep learning techniques. Hybrid approaches are made possible by the capacity to combine various neural network topologies into intricate ones within a single network.

The primary deep-learning techniques that surfaced throughout the examination of pertinent literature are briefly described here.

Artificial intelligence (AI) can deliver adaptable solutions for Internet of Things (IoT) systems by analyzing a variety of data types and quantities. By analyzing enormous volumes of IoT data, machine learning and data analytics are already being utilized to improve network performance and customer service. To spot potentially harmful behavior and stop anomalous activity, IoT networks can use behavioral analytics, anomaly detection, and pattern recognition. To characterize behaviors as “malware attack” or “normal”, this study presents a novel multi-layer architecture for an Internet of Things system that makes use of deep learning algorithms to forecast network data. This study makes use of two intrusion detection datasets—UNSW-NB15, KDDCup99, and IoT-23 datasets—which are commonly employed in machine learning studies on network data security. Because IoT datasets are high-dimensional, temporal, and multi-modal in nature, they pose considerable hurdles for analysis and detection. Compared to classic machine learning techniques, deep learning algorithms are more suited to complicated IoT datasets due to their huge processing capacity. Even while deep learning research on IoT security is still in its infancy, it has enormous promise for gleaning information from IoT data. The researchers think that IoT systems can be intelligently optimized using deep learning techniques. Recurrent neural networks, for instance, can process and store data at each stage before feeding it into the next because they can learn from prior time steps of incoming input. Regardless of the complexity of the neural network, hyperparameters can be changed to obtain lightweight functionality for Internet of Things solutions. This study aims to assess the efficacy of recurrent neural network techniques on intrusion detection datasets and investigate deep learning’s potential in cybersecurity and IoT. It is driven by the idea of applying deep learning to IoT network security. In the linked world of today, maintaining security necessitates evaluating enormous amounts of heterogeneous data—something that can only be completed with AI support.

Using real-time Internet of Things data, the researchers assessed several deep learning algorithms, such as recurrent neural networks, autoencoders, convolutional neural networks, and deep neural networks, to tackle this problem. The study issue finally led them to concentrate on recurrent neural networks because it required an algorithm that could learn from past data.

We evaluated various versions of gated recurrent unit (GRU), including bi-directional and multi-layer GRU, as well as CNN to devise efficient and lightweight solutions for the IoT network and obtain optimal results on the dataset. Most IDS-ML researchers utilize the UNSW-NB15, KDDCup99, and IoT-23 datasets for IoT network intrusion detection data. To make the data more suited for an IoT network, we layered it.

It has become impractical to rely on traditional intrusion detection systems (IDS) based on attack signatures to identify and block cyber-attacks against IoT infrastructures [3] due to the continual growth of cyber-attacks. While signature-based IDSs can accurately detect attacks that match pre-stored intrusion patterns, such as sequences of system calls or network traffic patterns, a problem arises when a new attack is discovered. Because signature-based IDSs require prior knowledge of an attack’s prospective signature, they are susceptible to new and unknown attacks. Traditional IDSs can only detect an attack if it already exists in their database, leaving them vulnerable to new and unknown threats. To address this limitation, anomaly-based intrusion detection systems (IDSs) have been proposed, which employ more intelligent methodologies than conventional IDSs. Anomaly-based IDSs establish a profile of “normal” behavior and then detect deviations, enabling them to detect potential new attacks (zero-day attacks). However, in a dynamic environment such as an IoT ecosystem, anomaly-based IDSs struggle to accurately detect all new intrusions, and the cost of false positives remains significant. Numerous zero-day assaults are undetected because of the limitations of IoT devices and conventional anomaly detection methods. Typically, critical defense mechanisms, such as a network intrusion detection system (NIDS), are used to analyze network traffic for abnormal behavior and facilitate such functions [4]. Assessing network traffic with anomaly-based IDSs can be used in conjunction with traditional cyber defense systems, such as firewall systems [5], to establish a secure environment and network. Using pre-trained models, anomaly-based IDSs can distinguish between benign and malicious traffic. This research paper is interdisciplinary, encompassing cyber security, artificial intelligence, and computer networks; consequently, a substantial quantity of effort has been devoted to comprehending the nuances of each subject. Our initial objective was to study the various attack categories present in an intrusion detection dataset. Then, we realized that deep learning techniques were required to classify the dataset for intrusion detection. In addition, we investigated IoT architecture and evaluated machine learning techniques that met IoT specifications. Signature-based methods, specification-based approaches, anomaly-based approaches, and hybrid strategies are the four primary categories of ID attacks [6]. Signature-based techniques entail comparing a set of network data to a feature database for similarities. If the scanned data match the database of signatures, they are deemed unlawful. This method is useful for precisely distinguishing the type of attack and permits system administrators to define rules and thresholds beforehand. The established criteria are then used to determine the current device and network status. If the threshold is exceeded or the rules are violated, the IDS will detect an abnormal condition and act accordingly [7].

The following are the main contributions of our paper.

1. An extensive analysis of pertinent studies in this field as well as an investigation of the importance of temporal and spatial characteristics in network data for ID intrusion detection.
2. The release of the DCGR_IoT model, which improves intrusion detection capabilities by integrating the temporal and spatial components of network data.
3. The creation of the DCGR_IoT model consists of two primary parts: a convolutional neural network (CNN)-based feature extraction module and a CGNN-based temporal feature extraction module. Notably, the DCGR_IoT improves the attention between local and global features as well as across various features by incorporating multi-object self-attention from the transformer architecture. Furthermore, to alleviate CNN’s restriction, a bidirectional memory network was integrated to preserve the temporal aspects of network traffic data.
4. Furthermore, to overcome CNN’s constraint in extracting temporal information, a bidirectional memory network was integrated to preserve the temporal properties of network traffic data.

Thorough testing of the suggested strategy using simulation tests was carried out with the Pytorch framework and Python programming language on the UNSW-NB15, KDDCup99, and IoT-23 datasets. Additionally, comparative experiments were performed, which showed that our model outperformed the other methods in terms of detection accuracy on both datasets. The effectiveness of building and refining large-scale intrusion detection systems (IDS) in an IoT environment is supported by these results.

The structure of this paper is as follows: Section 2 describes the fundamental architecture and related works for network intrusion detection. Our proposed technique, the deep complex gated recurrent network-based IoT network intrusion detection system (DCGR_IoT), is in Section 3. The fourth section introduces the verification and analysis of the efficacy and precision of the proposed model. Section 5 concludes our paper with a summary.

## 2. Background and Related Works

This section examines the critical role that cybersecurity plays in Internet of Things (IoT) systems. It does this by reviewing prior studies and acknowledging the advancements that have been made possible using machine learning (ML) and deep learning (DL) techniques. It highlights the necessity of intrusion detection systems (IDS) in IoT ecosystems by addressing the difficulties caused by the lack of these systems and stressing how vital it is to defend IoT networks. Even though there has been considerable progress in using ML and DL to create and implement IDS in IoT contexts, there is still a need for more research and development of intrusion detection techniques designed with the IoT landscape in mind.

This section meticulously evaluates the body of research on DL-based IDS intended for Internet of Things configurations, emphasizing gaps in the field’s current understanding. It becomes clear that a variety of cybersecurity issues call for creative solutions to successfully counter cyber threats and create security measures appropriate for the resource-constrained IoT network environment.

Technology breakthroughs have made IoT networks more significant, which has increased attention to security and intrusion detection systems.

The accuracy of intrusion detection in IoT networks is being improved by using ML and DL approaches in anomaly-based intrusion detection systems (IDS). Various IoT system layers present security challenges, including hardware manipulation, signal interference, and device limitations.

According to the paper [8], using machine learning techniques in SOA typically necessitates turning data into a set of features, or creating a feature space. It also highlights how crucial it is to choose the right features to maximize the accuracy of these techniques. So-called deep learning methods of neural networks are being employed more and more to handle challenges related to processing a high-power feature space, choosing an appropriate feature space, and several other problems. Compared to previous machine learning techniques, these methods improve the quality of anomaly detection for IoT networks and increase the automation of high-dimensional data processing. As of right now, there are no established guidelines for using deep learning techniques for anomaly detection.

Currently, deep learning techniques for addressing anomaly detection in IoT networks lack well-defined standards. The fact that the topic under discussion is still in its early stages of development establishes its applicability and validates the necessity of conducting a thorough examination of the techniques employed, categorizing, and contrasting them, and investigating current issues and potential solutions. The relevance of the task of classifying them is also determined by how diverse and variable the features of deep learning algorithms are when tackling the anomaly detection problem for IoT networks.

Because of the proliferation of dangerous data in cyber infrastructures and the growing sophistication and frequency of cyber-attacks, machine learning, data mining, and other interdisciplinary approaches are being used extensively to address cybersecurity concerns. These methods are used in many cybersecurity contexts, such as identification of signatures, identification of anomalies, scan identification, profiling of network traffic, and data mining that protects privacy.

One important use in this area is misuse detection, which is recognizing patterns of unauthorized conduct to anticipate and identify similar attempts in the future [9]. Fuzzy-rule-based techniques are used in cybersecurity systems to improve resilience and manage unpredictability.

These algorithms generate human-like decision-making competency, reducing the need for people to manually update the system as new threats occur [10]. Anomaly detection seeks to recognize any system event that deviates from a set of expected behaviors. According to some studies [11], an automated or semi-automatic strategy to detect unknown assaults on unlabeled data has higher potential, allowing cybersecurity specialists [12] to concentrate on the most likely attack data. ROC was used to evaluate the performance of random forests on the DARPA MIT KDDCup99 dataset [13]. When compared to previous unsupervised anomaly detection systems [14], the best detection rate was achieved by maintaining a low false positive rate; because of the involvement of numerous protocols, intrusion detection might take a significant amount of time and resources.

Machine learning entails developing models that can execute certain tasks by using relevant features. Machine learning methods are typically designed to address relatively specific problems with a limited number of feature types [15].

Deep learning, on the other hand, is a subfield of machine learning that uses several layers to represent input data to model complicated relationships among data. Each layer of a deep learning network is made up of neurons that can be connected to neurons on the same layer as well as neurons on other layers. The method used to make these connections differs based on the network type. A deep learning network’s layers compute and transform data, which is subsequently transmitted to the next layer [16].

Diro et al. [17] proposed utilizing deep learning to detect anomalies in IoT data and showed that it outperformed traditional IDSs in detecting coordinated IoT fog attacks. This study created an anomaly-based intrusion detection system that works in traditional networks, and they used the KDDCup99 dataset to train and evaluate their model [18]. The offered remedy was 95% accurate, and it should be applied. However, the KDDCup99 dataset used has non-uniform data and few unique records, making it challenging to generate trustworthy conclusions. An argument was made in favor of anomaly-based intrusion detection systems that rely solely on network features [19]. When employing several machine learning models, an R-tree technique produced the greatest results, with a 99.5% true positive rate and a 0.001% false positive rate. Their findings highlighted the usefulness of statistical techniques like random forest. Their dataset, however, is not a standard, raising validity difficulties. The method of identification for SDN compatibility consists of a signature-based ID and an anomaly [20]-based ID that is trained and tested on the NSL-KDD dataset. The detection accuracy was greater than 97.4%. On the other hand, intrusions discovered simply using anomaly detection cannot be separated from signature detection as well as the feature-limited anomaly-based SDN security architecture. The researchers compared the outcomes of various machine-learning methods. When it came to photo classification, a deep neural network with three hidden layers delivered a 76% level of accuracy. CNN-BiLSTM was used in [21] to solve the intrusion detection problem, with CNN concentrating on spatial features and BiLSTM on temporal features. To improve efficiency and maintain dataset balance, hybrid sampling—OSS with SMOTE—was utilized. All the compared models’ training times decreased after hybrid sampling. CNN-BiLSTM fared better for every other parameter, even though its training pace was slower than that of LeNet-5. Interestingly, the evaluation did not include the most recent datasets such as UNSW-NB15 and NSL-KDD.

In [22], a deep neural network (DNN) with three hidden layers outperformed traditional machine-learning techniques in intrusion detection. Ada Boost, decision tree, K-Nearest Neighbor, linear regression, Naive Bayes, random forest, SVM-Linear, and SVM-Rbf on the KDD were also evaluated in addition to DNN setups with one to five hidden layers. A novel hybrid intrusion detection system (IDS) is presented in [23] that utilizes a convolutional recurrent neural network (CNN-RNN) architecture for intrusion detection. In this case, the RNN records the temporal elements, whereas the CNN extracts the spatial information. Oversampling of cases from minority classes is necessary to balance the dataset. To improve generalization and reduce overfitting, layers with Gaussian noise were included before the CNN and RNN layers in the performance evaluation process.

In research [24], a CNN is used to approach intrusion detection after first converting data into an “image” matrix format. Batch normalization (BN) is used in conjunction with the principal component analysis (PCA) and autoencoder (AE) approaches to reduce the dimensionality of the feature space and optimize learning. Several RNN architectures (RNN, LSTM, and GRU) were investigated in [23] for intrusion detection; hyperparameter selection and experiments on the KDDCup99 and UNSW-NB15 datasets were performed. Interestingly, RNN architectures perform better than nonrecurrent networks in terms of type I errors (false positives); LSTM and GRU both perform comparably well in this regard. Owing to the variety of attacks on the UNSW-NB15 dataset, the performance was somewhat lower, emphasizing the need for real-world data testing.

In [25], a model for intrusion detection was presented that combined two neural networks: a Shallow Neural Network (S-NN) and a Deep-Optimized Neural Network (D-ONN). S-NN prioritizes simplicity and speed, whereas the D-ONN provides complexity at the expense of speed. Correlation analysis and entropy techniques were used in the feature selection to produce better results. A Deep-Optimized Neural Network model was developed for intrusion detection using the KDDCup99 dataset, a widely recognized benchmark in the field. The model achieved remarkable performance, with an accuracy of 98%, precision of 93%, recall of 93%, and an F1 score of 98%. These results demonstrate the model’s exceptional ability to accurately classify network traffic as normal or malicious. The deep architecture of the neural network allowed it to effectively learn complex patterns and relationships within the data, enabling it to detect intrusions with high precision and recall [26]. To improve the intrusion detection accuracy [27] presented the SFSDT+RNN model, which focuses on R2L and U2R attack types. A hybrid SFSDT algorithm that combines decision tree (DT) and sequential forward selection (SFS) methods is used for feature selection.

To analyze modern deep learning methods in the field of intrusion detection, a comparison of the methods discussed above was performed.

The findings are provided from an examination of pertinent research and reviews on using deep learning to detect intrusions. The most popular deep learning techniques are described, compared, and a categorization scheme is suggested. The current trends and results in detecting anomalous attacks in IoT networks using deep learning techniques are noted as shown in Table 1.

Several implementations and studies of the CNN+CGRN approach for IoT anomaly detection have been conducted, confirming its efficiency in detecting anomalies in sensor data.

A recent work published in [30,31] offered a CNN+GRN technique for detecting anomalies in IoT sensor data. The researchers used a dataset of temperature and humidity sensor readings from a building to identify anomalies, which correlated to anomalous changes in temperature or humidity levels. They compared the performance of their proposed CNN+GRN technique to that of many other machine learning methods, such as classic statistical models and deep learning models that did not include the GRN component. In terms of accuracy and F1-score measures, the CNN+CGRN (convolutional neural network + convolutional gated recurrent network) approach outperformed the other techniques.

The authors developed a CNN+CGRU technique for detecting anomalies in smart building time-series sensor data. The authors used a dataset of sensor readings from several sensors across the building to identify anomalies, which correlated to anomalous changes in the sensor readings. They compared the performance of their proposed CNN+CGRU approach to that of existing machine learning methods, such as classical statistical models and deep learning models that did not include the GRU component. The CNN+GRU strategy surpassed all other methods in terms of accuracy, precision, and recall, according to the data.

Overall, these results show that the CNN+CGRN strategy is successful for IoT anomaly detection and has the potential to increase the accuracy and efficiency of anomaly detection systems for IoT applications.

It is possible to streamline the feature construction stage by utilizing the novel deep neural learning-based intrusion detection system (DCGR_IoT), which was created especially for protecting bi-directional communication networks in Internet of Things environments. This, along with the similarity of the obtained metric values for the compared models, validates the use of DCGR_IoT for intrusion detection. CNN-based feature screening removes superfluous features, complex gated recurrent networks (CGRNs) create multi-space feature subsets for richer spatial representation, extract important features, and create feature associations to identify intrusions to evaluate the suitability of deep learning techniques for intrusion detection, combined, and the outcomes of its comparison with earlier models are created using deep learning.

## 3. The Deep Neural Learning-Based Approach for IoT Anomaly Detection (DCGR_IoT)

There are three phases in the DCGR_IoT strategy described in this section:

The first phase is preprocessing the dataset to prepare the dataset and meet the input requirements. The original characteristics must be reshaped and encoded using a multilayer convolutional neural network before being fed into the model. To keep the information flowing through the network and stop gradient fading, residual links were introduced.

The second phase for temporal-spatial feature extraction is CNN-assisted spatial feature extraction: CNN is utilized to acquire spatial characteristics and temporal feature extraction with CGRNs: multiread self-attention is employed with multiple transformer encoders to extract greater feature information. To obtain temporal feature representations among the features, the temporal relationship between the pre- and post-features was mined using several CGRNs blocks as mentioned Section 4.

The combining features and categorization in the third phase, the acquired spatial and temporal characteristics, were combined through concatenation for the classification of intrusion attacks. The categorization module receives the fused features. Using the SoftMax function, several completely connected layers were used to perform the final classification.

### 3.1. The Pre-Processing Dataset

The dataset is encoded and structured to comply with the model’s requirements after the transform has been adjusted. Next, the dataset is split into three sections: with the sets for training and validation, and testing configuration, the testing set is used to assess the model’s performance, whilst the training and validation sets are used to train the model.

After encoding and normalization, the dataset is prepared for DCGR_IoT model training. However, depending on the CNN model, further preparation is needed:

- A 1D vector must be created from the dataset to use it in a 1D CNN model.
- The processed data must be transformed into a matrix format for a 2D CNN model.
- With these changes, the data are guaranteed to be in the right format for any kind of CNN model.

### 3.2. Convolutional Neural Networks (CNN) for Feature Selection

**Figure 1:**
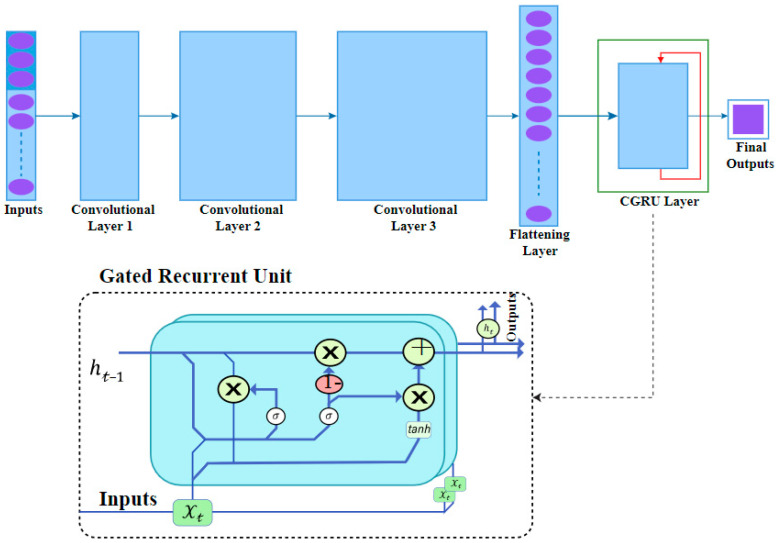
The architecture convolution neural network in DCGR_IoT.

The DCGR_IoT system mainly uses CNN for spatial feature extraction. This aids in separating crucial characteristics from irrelevant ones in network traffic, helping to remove unnecessary information. Not all layers in a convolutional neural network (CNN) are fully connected, and neurons have limited connections, being linked to only a tiny portion of the preceding layer. Convolutional layers that are stacked make it easier to abstract low-level characteristics into high-level features. A convolutional layer is made up of a sliding filter that is applied across the input data’s dimensions. The filter output is passed into a non-linear activation function, which produces activation maps. The network modifies the filters during operation so that activations happen when the filter passes over favorable traits, allowing it to learn and improve its performance [32]. The size of the filter in a convolutional layer can vary, although 3 × 3 is a popular size. A fully connected layer, also known as a dense or linear layer, is made up of neurons that are linked to all neurons in the layer above. These layers are used to build an ANN. The pooling layer subsamples the previous layer’s output, lowering dimensionality and the number of learned parameters. When paired with a convolutional layer, the pooling layer gives translation invariance to the network, as shown in Figure 1.

A dropout layer mitigates overfitting by deleting data at random with a specified probability. It also minimizes the preceding layer’s bias towards weights, and its effects are amplified when the dataset is small. The activation function f(x) in rectified linear units (ReLU) accelerates the network’s training process. The sparsity of the activation maps is also influenced by ReLU, in Equation (1).

(1) f(x)=max(x; 0)

Batch normalization is a technique for normalizing variables in relation to the current batch that can be applied to either direct inputs or prior layer activations. It speeds up network training, improves regularization, and lowers network generalization errors. Skip connections establish new connections between neurons in different levels that may not be connected sequentially. Skip connections improve network training and minimize linear dependence between neurons by disrupting the symmetry of the neural network, avoiding singularities in the loss landscape. Trainable parameters in a neural network are those that change during training, whereas nontrainable parameters do not change. By dividing the dataset depending on these proportions, a stratified split of a dataset retains the normal distribution of classes. Cisco NetFlow is a network flow representation protocol [29]. It contains data characteristics and bidirectional flow information retrieved from network packet headers that are unrelated to the content.

### 3.3. Complex Gated Recurrent Network

A more intricate and spatial representation of network traffic is made possible by the construction of multidimensional feature subsets using complex gated recurrent networks (CGRNs). This helps much more in locating and removing unnecessary data.

A gated recurrent network (GRN) is a sort of recurrent neural network (RNN) that uses gating methods to selectively transfer and update information across the network. Unlike typical RNNs, which use a simple recurrent connection between hidden units, GRNs govern the flow of input using more complicated gating units.

Cho et al. [33] introduced the gated recurrent unit (GRU) as the most popular architecture for GRNs in 2014. To govern information flow, GRUs employ two gating units: the update gate and the reset gate. The update gate decides how much of the prior hidden state to keep and how much of the new input to add, whereas the reset gate decides how much of the previous hidden state to forget.

**Figure 2:**
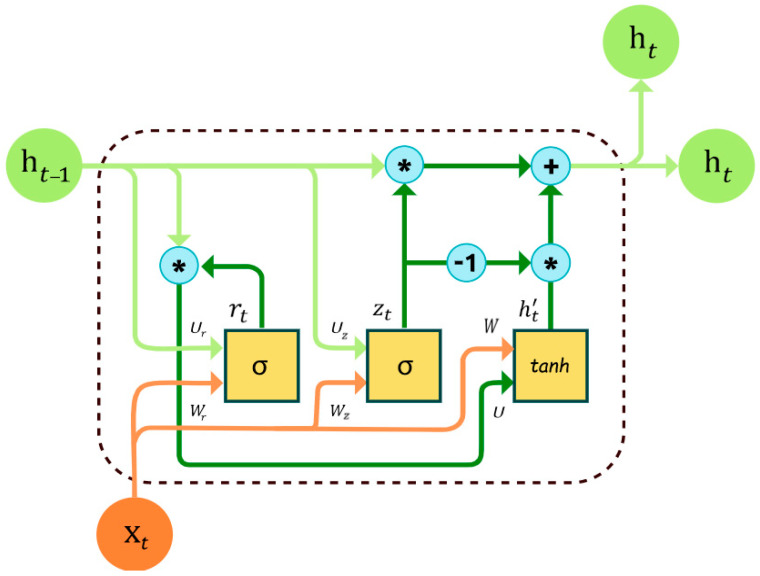
The architecture of complex gated recurrent network.

Another widely used GRN architecture is the Long Short-Term Memory (LSTM) network, which was first defined by Hochreiter and Schmidhuber in 1997. LSTMs, like GRUs, use gating mechanisms to selectively send and update data across networks, but their architecture is more complex, with separate input, output, and forget gates. GRNs are especially useful for modeling long-term dependencies because they allow the network to selectively recall and forget information over time, as seen in Figure 2. They have been used in a variety of fields, such as natural language processing, speech recognition, and image captioning.

Deep complex gated recurrent networks (Deep Complex GRUs) combined with convolutional neural networks (CNN) can be used to create a powerful model for processing complex-valued data and capturing spatial and temporal dependencies. In this setup, the CNN is used for feature extraction, while the Deep Complex GRU processes the extracted features and captures temporal dependencies. Here is a description of the combined model with equations:

#### 3.3.1. CNN for Feature Extraction

Equation (2) represents a 2D convolution operation, where the input feature map is convolved with the convolution filter, and the result is passed through an activation function to produce the output feature map.

- Convolutional layer:
  (2) $$y_{i,j}=\alpha \left(\sum_{m=0}^{M-1}\;\sum_{n=0}^{N-1}\;w_{m,n}\cdot x_{i+m,j+n}+b\right)$$
  In Equation(2), where $x_{i,j}$ is the input feature map, $w_{m,n}$ are the convolutional weights of m×n and b: Bias term. $y_{i,j}$: Output feature at location (i,j) in the output feature map. α: Activation function (typically a non-linear function such as ReLU, tanh, or sigmoid).$x_{i+m,j+n}$: Input feature values at locations (i+m,j+n) in the input feature map.
- Pooling layer:
  The equation describes the max pooling operation in a neural network. The max pooling operation is used to down sample the input feature map by selecting the maximum value within a specified window size. The equation is as follows:
  (3) $$y_{i,j}=\underset{m=0}{\max}k-1\underset{n=0}{\max}l-1x_{i+m,j+n}$$
  In Equation (3), where $y_{i,j}$ is the output feature map, and the max operation is performed over a specified window size (e.g., 2 × 2), $y_{i,j}$ is the output feature value at location (i,j) in the output feature map after pooling. $x_{i+m,j+n}$: Input feature values within the pooling window, where m and n range over the dimensions of the pooling window of size k∗l, where k: Height of the pooling window and l: Width of the pooling window.
- Flattening:
  The output feature maps from the CNN to create a 1D vector that can be fed into the Deep Complex GRU.

#### 3.3.2. Deep Complex GRU for Processing Extracted Features and Capturing Temporal Dependencies

- Update Gate:
  The update gate controls how much of the previously hidden state $h_{t-1}$ is carried forward to the current time step in Equation (4). It uses the sigmoid activation function to squash the value between 0 and 1.
  (4) $$z_{t}=\sigma \left(W_{z}*x_{t}+U_{z}*h_{t-1}+b_{z}\right)$$
- Reset Gate:
  The reset gate controls how much of the previous hidden state $h_{t-1}$ is forgotten or “reset” at the current time step. It also uses the sigmoid activation function in Equation (5).
  (5) $$r_{t}=\sigma \left(W_{r}*x_{t}+U_{r}*h_{t-1}+b_{r}\right)$$
- Candidate Hidden State:
  The candidate hidden state ${\tilde{h}}_{t}$ in Equation (6) is an intermediate representation computed at each time step in a gated recurrent neural network for GRU. It represents the new information that could potentially be added to the hidden state.
  (6) $${\tilde{h}}_{t}=\tanh\left(W*x_{t}+r_{t}*\left(U*h_{t-1}\right)+b\right)$$
- Hidden State:
  (7) $$h_{t}=\left(1-z_{t}\right)*h_{t-1}+z_{t}*{\tilde{h}}_{t}$$
  The hidden state $h_{t}$ is the final output of a gated recurrent neural network at time step t. It is computed as a weighted combination of the previous hidden state t−1 and the candidate hidden state $h_{t}$.
  $z_{t}$: The update gate value at time step t−1
  $h_{t-1}$: The previous hidden state
  $h_{t}$: The candidate hidden state at time step t

The update gate controls how much of the previous hidden state $h_{t-1}$ is carried forward and how much of the new candidate hidden state $h_{t}$ is incorporated. When $z_{t}$ is close to 0, the hidden state $h_{t-1}$ will be mostly determined by the previously hidden state $h_{t-1}$. When $z_{t}$ is close to 1, the hidden state will be mostly determined by the candidate’s hidden state $h_{t}$.

In these equations, the variables are defined as follows:

$x_{t}$: Input at time step t (extracted features from the CNN)

$h_{t}$: Hidden state at time step t

$h_{t-1}$: Hidden state at time step t−1

W, U,$\;W_{z}$,$\;U_{z}$,$\;W_{r}$, $U_{r}$: Weight matrices

b,$\;b_{z}$, $b_{r}$: Bias terms

*: Complex-valued matrix multiplication

σ: Sigmoid activation function

tanh: Hyperbolic tangent activation function

By combining CNN for feature extraction and Deep Complex GRU for processing and capturing temporal dependencies, the model can effectively handle complex-valued data and provide a powerful solution for various applications, such as IoT network intrusion detection systems.

Complex gated recurrent networks (CGRNs) are an extension of the gated recurrent unit (GRU) model, designed to handle complex-valued data, which is particularly useful in scenarios where phase information is critical, such as in signal processing or communication systems. In the context of DCGR_IoT, CGRNs are used to construct multi-space feature subsets and to extract crucial features for intrusion detection.

Let $h_{t}$ represent the hidden state at time t, and $x_{t}$ represents the input at time t. The CGRN updates its hidden state using two gates: the reset gate $r_{t}$ and the update gate $z_{t}$.

The reset gate determines how much past information to forget. It is computed as follows:

In the context of complex-valued data, the complex gated recurrent network (CGRN) updates its hidden state using two gates: the reset gate $r_{t}$ and the update gate $z_{t}$. Here is how the computations are adapted for complex-valued data:

- Reset Gate $r_{t}$:
  (8) $$r_{t}=\sigma \left(W_{xr}\cdot x_{t}+W_{hr}*h_{t-1}+b_{r}\right)$$
  where σ is a complex-valued activation function (e.g., complex sigmoid or complex softmax), $W_{xr}$ and $W_{hr}$ are complex-valued weight matrices for the input and hidden states, respectively, and $b_{r}$ is a complex-valued bias term.
- Update Gate $z_{t}$:
  (9) $$z_{t}=\sigma \left(W_{xz}\cdot x_{t}+W_{hz}*h_{t-1}+b_{z}\right)$$
  with $W_{xz}\;W_{hz}$ and being complex-valued weight matrices, and $b_{z}$ being a complex-valued bias term.
- The Hidden State Update where the new memory content $n_{t}$ is computed by combining the reset gate with the previous hidden state and the current input: New Memory Content $n_{t}$:
  (10) $$n_{t}=tanh\left(W_{xn}\cdot x_{t}+r_{t}*\left(W_{hn}*h_{t-1}\right)+b_{n}\right)$$
  where tanh is replaced with a complex-valued hyperbolic tangent function, $W_{xn}$ and $W_{hn}$ are complex-valued weight matrices, and $b_{n}$ is a complex-valued bias term. The element-wise multiplication ∗ is performed in the complex domain.
- Final Hidden State $h_{t}$:
  (11) $$h_{t}=\left(1-z_{t}\right)*h_{t-1}+z_{t}*n_{t}$$

Here, the element-wise multiplication ∗ and addition are performed in the complex domain. The final hidden state $h_{t}$ is also complex-valued.

By adapting these computations to the complex domain, CGRNs can effectively process complex-valued data, capturing both the magnitude and phase information. This makes them suitable for applications where complex-valued data are inherent, such as in signal processing, communication systems, and certain machine-learning tasks involving complex features.

In the context of complex-valued data, all operations are performed in the complex domain. The sigmoid function is replaced with a complex-valued activation function, and the weight matrices and bias terms are complex-valued as well. The element-wise multiplication and addition operations are also performed in the complex domain.

The CGRN’s ability to handle complex-valued data allows it to capture phase information and construct richer feature representations, which is crucial for identifying intricate intrusion patterns in IoT networks.

The combination of convolutional neural networks (CNNs) [34] and gated recurrent networks (GRNs) has been used in IoT anomaly detection applications to effectively capture both spatial and temporal features in sensor data.

The CNN is utilized in this approach to extract spatial characteristics from sensor data [35], while the GRN is used to model temporal dependencies in the data. CNN is commonly employed as the model’s front end, with numerous convolutional layers followed by pooling and normalization layers. The CNN output is then passed into the GRN, which processes the feature map sequence generated by the CNN. The GRN can be implemented using various architectures of GRUs which are used to model the temporal dependencies in the data. The output of the GRN is then fed into a fully connected layer, which performs classification to detect anomalies in the data.

**Figure 3:**
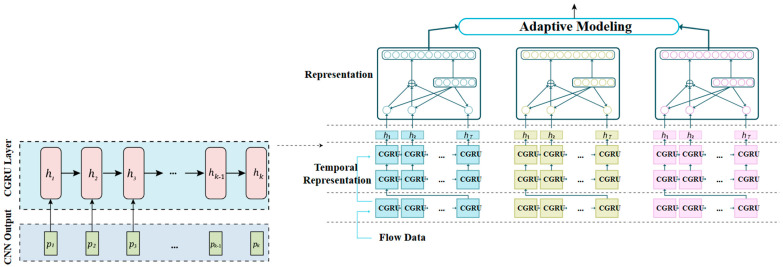
The architecture proposes DCGR_IoT.

One advantage of using CNNs and GRNs is that they can handle high-dimensional sensor data and capture complex patterns in the data. Additionally, the use of GRNs allows the model to effectively handle sequences with long-term dependencies, which is important for anomaly detection in IoT applications where anomalies may occur over a long period of time as shown in Figure 3.

Overall, the combination of CNNs and GRNs has shown promising results for IoT anomaly detection applications and has the potential to improve the accuracy and efficiency of anomaly detection systems.

To evaluate the DCGR_IoT system on UNSW-NB15, KDDCup99, and IoT-23 datasets, the steps for the DCGR_IoT A approach are as follows.

- Phase 1: Data preprocessing: Loading and preprocessing the UNSW-NB15, KDDCup99, and IoT-23 datasets. This may include normalizing data, handling missing values, and converting categorical variables to numerical representations. Ensure that the data are consistent with the Deep Complex GRU model.
- Phase 2: Feature Extraction: Extract relevant features from pre-processed datasets using convolutional neural networks (CNN) as mentioned in the DCGR_IoT system. This step helps in selecting important features and filtering out unnecessary data to improve computational efficiency.
- Phase 3: Model Training: Train the Deep Complex GRU (CGRN) model on the extracted features using a labeled dataset containing examples of normal and malicious network traffic. The model will learn to recognize patterns and temporal dependencies in the data that indicate intrusion attempts.
- Phase 4: Model Evaluation: Evaluate the performance of the DCGR_IoT system using a separate validation dataset for both the UNSW-NB15, KDDCup99, and IoT-23 datasets. This step helps ensure that the model generalizes well to unseen data and can effectively detect intrusions.
- Final stage:

Performance metrics: Calculate the detection accuracy, precision, recall, and F1 score of the DCGR_IoT system on the two datasets. Compare the results with other modern intrusion detection systems to prove the effectiveness of the proposed system.

Analysis: Analyze the results and identify any potential areas for improvement or further research. This may include exploring different architectures, hyperparameters, or feature extraction techniques to improve the performance of the DCGR_IoT system.

By following these steps, you can evaluate the DCGR_IoT system on the UNSW-NB15, KDDCup99, and IoT-23 datasets and demonstrate its potential as an effective solution for defending IoT networks against sophisticated cyberattacks. The high detection accuracy of 99.2% mentioned in the description indicates that the DCGR_IoT system can be a promising approach for IoT network intrusion detection.

1. Preprocessing: The sensor data are preprocessed to fill in missing values and normalize the data.
2. Feature selection: The preprocessed sensor data are fed into a CNN to extract spatial features from the sensor data. Feature selection in the proposed framework for intrusion detection involves identifying and selecting the most important features from the dataset to improve model performance and reduce computational costs. The method used for feature selection is a correlation-based filter approach, where features are chosen based on their correlation scores. Features with a correlation score higher than a specified threshold are considered highly correlated, and redundant features are removed to streamline the dataset. This process not only enhances the model’s accuracy but also decreases the training time by reducing the number of input variables. Once the dataset was normalized, we used feature selection algorithms to identify significant features. Among the different possibilities, we used the correlation-based filter method. This method improves storage economy while reducing computation time. We chose features according to their correlation scores by applying a correlation-based filter approach. We used the Pearson correlation approach, as shown in Equation (2) to evaluate the correlations between the attributes. Features with values close to −1 or 1 are highly correlated, whereas those near 0 are uncorrelated, according to the Pearson correlation coefficient, which ranges from −1 to 1. Features with high correlation values were considered redundant. We handle this by designating features as redundant if their correlation values are greater than a certain threshold, which we set as 0.95. Therefore, we eliminated one feature from each group of redundant features. During model building, we applied correlation for feature selection in UNSW-NB15, KDDCup99, and IoT-23 datasets’ features.
3. Sequence generation: The output of the CNN is then fed into a GRU to generate a sequence of feature maps, which capture the temporal dependencies in the data.
4. Classification: The output of the GRU is then fed into a fully connected layer, which performs classification to detect anomalies in the data.
5. Training: The DCGR_IoT are trained on a labeled dataset of sensor data, using backpropagation and gradient descent to optimize the model parameters.
6. Evaluation: The performance of the DCGR_IoT model is evaluated using various metrics, such as accuracy, precision, recall, and F1-score.
7. Deployment: The trained model is deployed in a real-world IoT environment, where it continuously monitors sensor data and detects anomalies in real time.

## 4. Implementations and Experiments

**Figure 4:**
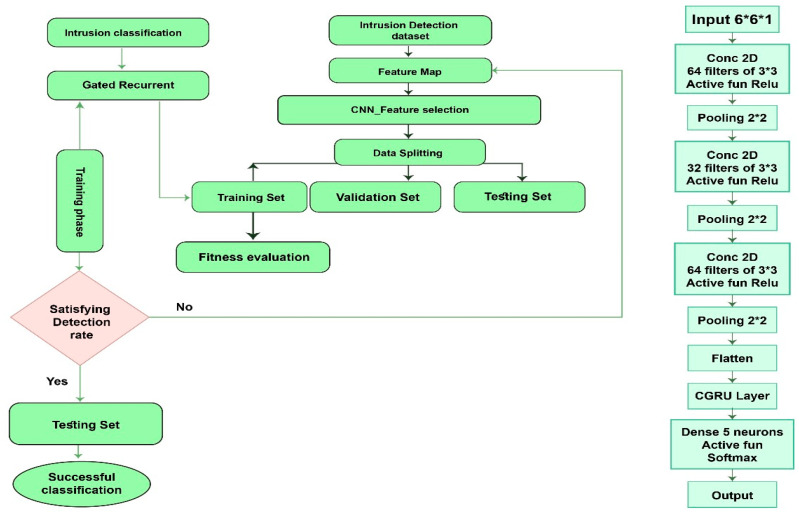
The flowchart proposes Model.

In this section, we showcase the DCGR_IoT model for conducting anomaly detection experiments on an IoT dataset. We assess the model’s performance in detecting anomalies within IoT network intrusion detection systems and compare it with other widely used anomaly detection algorithms to highlight its superiority. Furthermore, to validate the effectiveness of our proposed improvement method, we perform ablation experiments, comparing and analyzing the model’s anomaly detection performance before and after the enhancements. The outcomes of these experiments serve to further confirm the feasibility and effectiveness of our DCGR_IoT model and its improved method in practical applications. The experiments utilized Python’s TensorFlow library. After conducting multiple experimental validations, the model hyperparameters were set as follows: learning rate = 0.001, optimizer = SGD, and number of epochs = 100, Weight Decay: 0.0001, Dropout Rate: 0.01. Hyper Parameter tuning in DCGR_IoT model is CNN1 (Convolution layers 64 filters 3 × 3), CNN1 (Convolution layers 32 filters 3 × 3) GRU1 and GRU1 Hidden Units [64, 128], The Dropout [0.0, 0.1], The Activation “relu”as shown in Figure 4.

The following hardware configuration was used for the comparative studies and experimentation for the intrusion detection model presented in this paper: Operating System: Windows 64-bit, Processor: 2.80 GHz Intel Core i7 CPU, 16 GB of RAM for graphics processing unit: Python-compatible Nvidia GPU with 4 GB of RAM. All of the experimental and comparative tests covered in this paper were conducted using this setup.

### 4.1. Evaluation Metrics

The optimal metric for IoT anomaly detection is decided by the specific situation and the system’s goals. Different measures have different strengths and weaknesses, and the best metrics to employ are determined by the most important component of system performance. If the goal is to detect as many anomalous cases as possible, recall, which measures the proportion of true positive predictions out of all real anomalous instances in the dataset, may be the most relevant parameter. A high recall shows that the model can discover a significant fraction of the anomalous cases in the sample.

If the goal is to reduce false positives (occurrences that are anticipated to be abnormal but are normal), accuracy may be the most relevant metric. A high precision suggests that the model can anticipate anomalous cases effectively while minimizing false positives.

F1-score may be an appropriate metric to utilize if a balance between recall and precision is sought. The F1-score in Equation (15) is a harmonic mean of precision and recall in Equation (12), with equal weight given to both metrics.

AUC-ROC can provide a measure of the model’s overall performance in discriminating between anomalous and normal occurrences, and the confusion matrix can provide a thorough breakdown of the classification findings.

The best metric for detecting IoT anomalies is determined by the situation and the system’s aims. Distinct metrics have distinct strengths and limitations, and the optimal one to use is determined by the most critical aspect of system performance. The confusion matrix is a table that summarizes the results of an instance classification task, such as IoT anomaly detection. The number of true positives (TP), true negatives (TN), false positives (FP), and false negatives (FN) for each class (anomalous and normal) is shown in the confusion matrix.

The confusion matrix can be used to compute metrics like accuracy, precision, recall, and F1-score.

The accuracy is defined as (TP + TN)/(TP + TN + FP + FN)(12)

Precision = (TP + FP)(13)

TP = (TP + FN)(14)

F1-score = 2 ∗ (precision ∗ recall)/(precision + recall)(15)

The confusion matrix can be used in IoT anomaly detection to evaluate the performance of a classification model in recognizing anomalous instances from sensor data. True positives are instances that are correctly classified as anomalous, true negatives are instances that are correctly classified as normal, false positives are instances that are incorrectly classified as anomalous (normal instances classified as anomalous), and false negatives are instances that are incorrectly classified as normal (anomalies classified as normal). By analyzing the confusion matrix, it is possible to determine the classification model’s strengths and shortcomings and alter its parameters to improve its performance. For example, if the model has a high false positive rate, the classification threshold may need to be adjusted or more features added to the model to increase its capacity to distinguish between anomalous and typical cases. Overall, the confusion matrix is a good tool for evaluating classification model performance in IoT anomaly detection.

### 4.2. Performance Evaluation UNSW-NB15 Dataset

**Figure 5:**
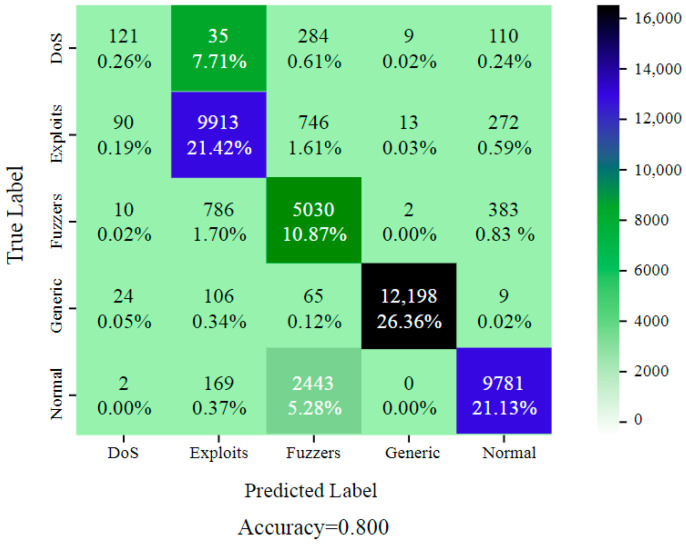
DCGR_IoT confusion matrix for UNSW-NB15 dataset.

**Figure 6:**
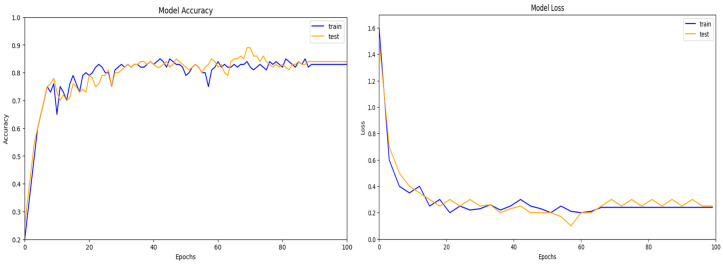
DCGR_IoT training and testing performance on UNSW-NB15 dataset.

Dataset UNSW-NB15 includes records of both benign traffic and five different types of attacks, such as Fuzzers, Normal, Generic, DoS, and Exploits. It was developed by the Australian Centre for Cyber Security (ACCS). The selected features determined the optimal performance of the model (DoS, Exploits, Fuzzers, Normal and Generic). Furthermore, when the entire feature set was used to train the intrusion detection system (IDS) model on this dataset, it achieved an accuracy of 80.00%, as shown in Figure 5. The records were gathered from three real-world websites: BID (Symantec Corporation), CVE (Common Vulnerabilities and Exposures), and MSD (Microsoft Corporation) (Microsoft Security Bulletin). The results for the UNSW-NB15 dataset are presented in Figure 6. It was found that three layers of CNN achieved the highest accuracy. For the final feature selection testing, DCGR_IoT was utilized. 

### 4.3. Performance Evaluation DCGR_IoT a Using the KDDCup99

KDDCup99 is a well-known dataset for intrusion detection research that was generated for the 1999 KDD Cup data mining competition. Researchers at the University of California, Irvine generated the dataset, which contains network traffic data taken from a simulated network environment.

**Figure 7:**
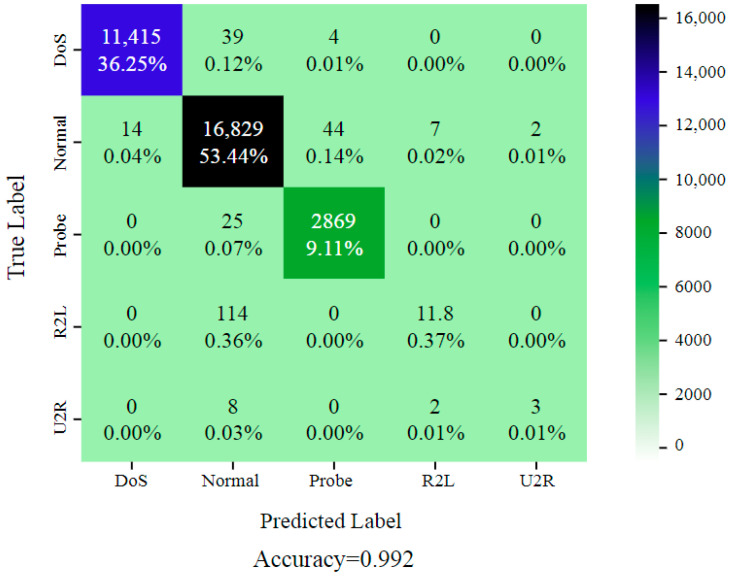
DCGR_IoT is a confusion matrix for the KDDCup99 dataset.

**Figure 8:**
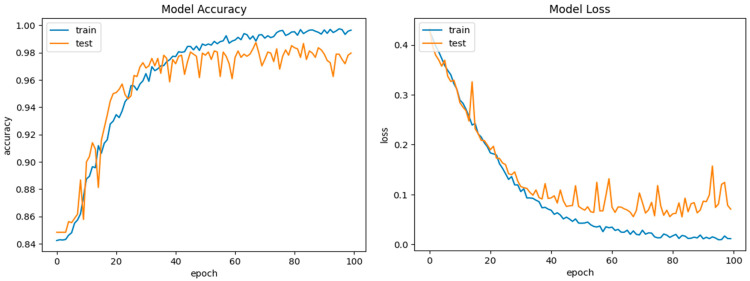
DCGR_IoT training and testing performance on KDDCup99 dataset.

The KDDCup99 dataset comprises both regular and malicious traffic, as well as 41 network traffic features such as protocol type, service, source and destination IP addresses, and connection length. There are four types of attacks in the dataset: DoS (Denial of Service), Probe, R2L (Remote-to-Local), and U2R (User-to-Root). The attacks were created with a variety of tools and approaches, and the dataset includes raw network traffic data as well as preprocessed CSV files for each attack scenario. The KDDCup99 dataset has been frequently utilized in research projects to assess the efficacy of machine learning algorithms in identifying network intrusions. However, the dataset has some drawbacks, including the fact that it is based on a simulated environment and contains outdated attack methodologies and traffic patterns. Because of these constraints, newer and more diverse datasets for intrusion detection studies have been generated throughout time. Nonetheless, the KDDCup99 dataset is still a popular resource for network security researchers and practitioners. The KDDCup99 dataset is open to the public and can be obtained from the dataset’s official website: http://kdd.ics.uci.edu/databases/kddcup99/kddcup99.html (accessed on 2 August 2024). The selected features determined the optimal performance of the model (DoS, U2R, R2L, Normal, and Probe). The technique was validated with 496,020 training records, 311,028 testing records, and a 99.2% total accuracy as shown in Figure 7. To assess it further, a confusion matrix was applied. Figure 8 shows the training and validation accuracies.

### 4.4. Performance Evaluation DCGR_IoT Using the IoT-23 DATASET

**Figure 9:**
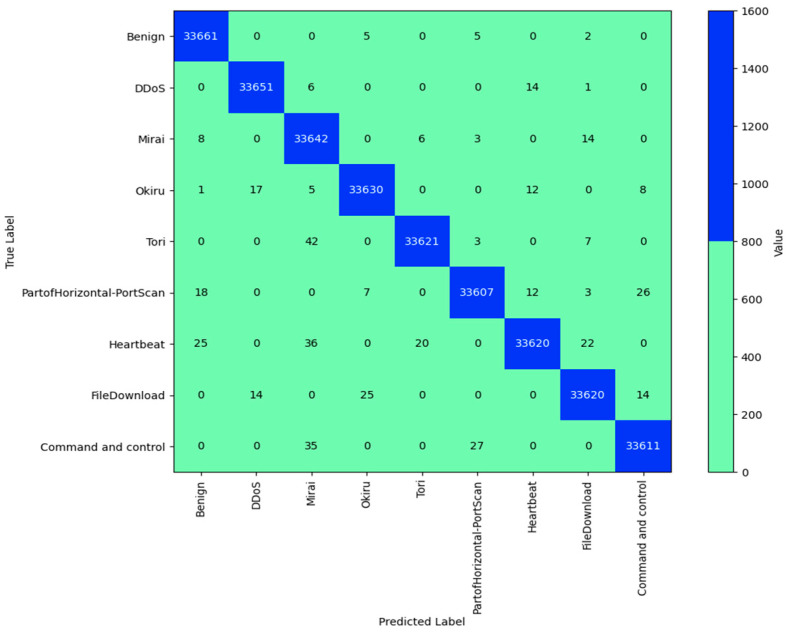
DCGR_IoT confusion matrix for IoT-23 dataset.

The IoT-23 dataset includes both benign and malicious traffic, simulating an IoT network in a smart house. To simulate benign circumstances, the network traffic gathered from three IoT devices was included. The dataset also includes various attacks, including DDoS attacks (https://www.stratosphereips.org/datasets-iot23 (accessed on 2 August 2024)). The confusion matrix for the performance of the model on the IoT-23 dataset is shown in Figure 9. A table called a confusion matrix is frequently used to explain how well a classification model performs when applied to a set of test data whose real values are known. The anticipated labels are shown in the columns, and genuine labels are shown in the rows. Each cell in the matrix represents the number of times a true label is applied to a predicted label.

‘Benign’, ‘DDoS’, ‘Mirai’, ‘Okiru’, ‘Tori’, ‘PartofHorizontal-PortScan’, ‘Heartbeat’, ‘FileDownload’, and ‘Command and control’ are the labels assigned to the rows and columns in this confusion matrix. The genuine label for each row and the anticipated label for each column were matched.

The number of times the model accurately predicted ‘Benign’ traffic is indicated in the cell where the ‘Benign’ row and ‘Benign’ column overlap. Comparably, the number of times that ‘DDoS’ traffic was incorrectly projected as ‘Mirai’ is indicated in the cell where the ‘Mirai’ column crosses the ‘DDoS’ row.

The diagonal of the matrix shows the model’s correct predictions from top left to bottom right. Misclassifications were represented as off-diagonal cells. The number of instances that a cell represents increases with deeper hue. The accuracy of the predictions of the classification model is known. This is determined by dividing the total number of forecasts by the number of accurate estimates. The accuracy in this instance is 0.9985384650914202, which is extremely near to 1. This suggests that the model predicts the right labels for the cases in the IoT-23 dataset with a high degree of accuracy. This paper examines the integration of current security mechanisms to improve the overall security of the DCGR_IoT model’s overall security:

All network traffic between IoT devices and the DCGR_IoT system can be encrypted using TLS integration. This will prevent network traffic from being intercepted or tampered with while it is being transmitted. 1. To impose end-to-end encryption, the system can be set up to allow TLS connections. 2. Digital signatures and standards, such as X.509 certificates, can be used to authenticate IoT devices. This ensures that only approved devices can send network traffic and connect to the system. 3. To verify device IDs prior to allowing traffic, the system can incorporate device authentication. To identify and address security events, security monitoring systems such as Security Information and Event Management (SIEM) can be incorporated. This will facilitate quick incident response and give real-time visibility into system security. The DCGR_IoT system’s overall security will be greatly improved by integrating these current security mechanisms. There will be defenses in place to protect the system from threats and attacks. To guarantee smooth integration and compatibility, meticulous preparation and configuration are necessary. All things considered, the system and its data are well protected by this multilayered security technique. We only consider packets that belong to IPv4 and one of the three protocols: TCP, UDP, or ICMP. The DCGR_IoT model has achieved the highest accuracy of 0.9985384650914202, 99.85% on the IoT-23 dataset. With an accuracy of 0.9985384650914202, 99.85% of the dataset’s instances were accurately predicted by the model. Given the extremely high accuracy, it appears that the model is doing a good job with this task.

**Figure 10:**
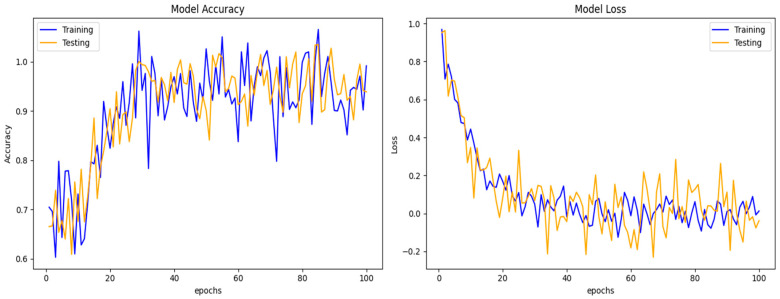
DCGR_IoT A training and testing performance on IoT-23 dataset.

Figure 10 shows the simulation results. DCGR_IoT A training and testing performance on the IoT-23 dataset presented two line plots, one depicting the model accuracy and the other showing the model loss over 100 epochs. The x-axis in both plots represents the number of epochs, and the y-axis indicates the respective metrics. The model accuracy shows that the training accuracy line generally exhibits an upward trend, suggesting that the model is learning and improving its performance on the training data over time. There are some fluctuations in the training accuracy, which are common during the training process. Additionally, there was a rising trend in the testing accuracy line, suggesting that the model’s generalization performance improved. In general, the training loss line trends downward, showing that the model learns to reduce the error between its predictions and the actual values. The training loss shows variations similar to those in the accuracy plot. The testing loss line likewise exhibits a downward trend, indicating that the model is becoming more effective for data that have not yet been observed. The model learns effectively, as both training and testing accuracy increase, while training loss decreases.

## 5. Conclusions

As the period of employing IoT network intrusion detection systems and other areas in real-life approaches, this technique can be linked with IoT situations as well. As we have highlighted in previous works [23,24,25,26,27,28], one of the directions of the modern technology and industrial revolution is IoT. We also examined the differences and parallels between IoT network intrusion detection approaches. One of the intrinsic new abilities of this intrusion detection technology is its security aspects. One of the primary drawbacks of current IoT solutions is the centralized system. The rapid expansion of the Internet of Things (IoT) has underscored the critical need for robust network security measures. Traditional intrusion detection systems (IDS) struggle to adapt to the unique challenges posed by IoT environments, including the diverse range of devices, complex network traffic patterns, and the imperative for real-time detection capabilities. To tackle these issues, we introduce DCGR_IoT, a cutting-edge intrusion detection system (IDS) based on deep neural learning designed to safeguard bidirectional communication networks in IoT settings. DCGR_IoT leverages advanced techniques to enhance anomaly detection capabilities. It employs convolutional neural networks (CNN) for spatial feature extraction and filters out redundant data to improve computational efficiency. Additionally, complex gated recurrent networks (CGRNs) are utilized in the temporal feature extraction module, which is a key component of DCGR_IoT. Moreover, DCGR_IoT utilizes complex gated recurrent networks (CGRNs) to create multidimensional feature subsets, enabling a more comprehensive spatial representation of network traffic and facilitating the extraction of crucial features vital for intrusion detection. The efficacy of DCGR_IoT was validated through extensive testing on the UNSW-NB15, KDDCup99, and IoT-23 datasets, achieving a high detection accuracy of 99.2%. These findings highlight the potential of DCGR-IoT as a potent solution for protecting IoT networks against advanced cyber threats.

## Figures and Tables

**Table 1:** Comparison of deep learning techniques for intrusion detection from an examination of pertinent research and reviews.

Ref.	Model	Dataset	Accuracy, Precision, Recall, F1
[21]	CNN-BiLSTM	NSL-KDD	F1 = 85.14, Recall = 84.49, Precision = 85.82, Accuracy = 83.58
[21]	CNN-BiLSTM	DARPA1998	Accuracy = 99.68
[22]	DNN	KDDCup99	F1 = 95.5, Recall = 91.5, Precision = 99.7, Accuracy = 93
[23]	(CNN-RNN)	CSE-CIC-IDS 2018	F1 = 0.976, Recall = 0.9712, Precision = 0.9633, Accuracy = 97.75
[24]	CNN	KDDCup99	Accuracy = 94
[25]	(RNN, LSTM, GRU)	UNSW-NB15	RNN: F1 = 91.8, Recall = 96.5, Precision = 87.6, Accuracy = 88.3, Loss = 0.25 LSTM: F1 = 92.9, Recall = 97.3, Precision = 88.9, Accuracy = 89.9, Loss = 0.22 GRU: F1 = 92.8, Recall = 97.3, Precision = 88.6, Accuracy = 89.7, Loss = 0.23
[26]	Deep-Optimized Neural Network Model	KDDCup99	Accuracy = 98, Precision = 93, Recall = 93, F1 = 98
[27]	SFSDT + RNN (RNN, LSTM, GRU)	NSL-KDD	RNN: Accuracy = 89.6 LSTM: Accuracy = 92 GRU: Accuracy = 91.8
[28]	Adversarial Autoencoders (AAE) and Bidirectional Generative Adversarial Networks (BiGAN)	IoT-23	Accuracy = 95
[29]	2D Convolutional Neural Networks (2D-CNN)	IoT-23	Accuracy = 99.34

## Data Availability

The KDDCup99 dataset is open to the public and can be obtained from the dataset’s official website: http://kdd.ics.uci.edu/databases/kddcup99/kddcup99.html (accessed on 2 August 2024).
